# CRISPR/Cas9-Mediated Biallelic Knockout of IRX3 Reduces the Production and Survival of Somatic Cell-Cloned Bama Minipigs

**DOI:** 10.3390/ani10030501

**Published:** 2020-03-17

**Authors:** Xiangxing Zhu, Yanyan Wei, Qunmei Zhan, Aifen Yan, Juan Feng, Lian Liu, Dongsheng Tang

**Affiliations:** 1 Guangdong Provincial Engineering and Technology Research Center for Gene Editing, School of Medical Engineering, Foshan University, Foshan 528225, China; yanaifen@mail3.sysu.edu.cn (A.Y.); fengjuan0220@126.com (J.F.); llian2004@163.com (L.L.); 2 Guangdong Provincial Key Laboratory of Animal Molecular Design and Precise Breeding, School of Life Science and Engineering, Foshan University, Foshan 528225, China; wei_yanyan@126.com (Y.W.); qmzhan8@163.com (Q.Z.)

**Keywords:** CRISPR/Cas9-mediated gene editing, Iroquois homeobox 3 (*IRX3*), obesity, Bama minipig, somatic cell cloning

## Abstract

**Simple Summary:**

Bama minipigs are a local pig breed that is unique to China and possess several negative features, including high fat content, low feed utilization rate, and slow growth rate. The iroquois homeobox 3 (*IRX3*) gene has been implicated in human obesity and controls body mass and composition in mouse. In this study, we successfully generated *IRX3* biallelic knockout Bama minipigs using CRISPR/Cas9-mediated gene editing combined with somatic cell cloning. The results show that the use of *IRX3*^-/-^ cells as donor cells for the production of somatic cell-cloned pigs induces a significant decrease in the average live litter size and a significant increase in the average number of stillbirths. Moreover, the birth weight of surviving *IRX3*^-/-^ somatic cell-cloned pigs is significantly lower, and viability is poor such that all piglets die shortly after birth. Therefore, the preliminary results of this study suggest that *IRX3* may have important biological functions in pigs, and *IRX3* should not be used as a gene editing target to reduce fat content in Bama minipigs.

**Abstract:**

Bama minipigs are a local pig breed that is unique to China and has a high development and utilization value. However, its high fat content, low feed utilization rate, and slow growth rate have limited its popularity and utilization. Compared with the long breeding cycle and high cost of traditional genetic breeding of pigs, clustered regularly interspaced short palindromic repeats (CRISPR)/CRISPR-associated (Cas) endonuclease 9 system (CRISPR/Cas9)-mediated gene editing can cost-effectively implement targeted mutations in animal genomes, thereby providing a powerful tool for rapid improvement of the economic traits of Bama minipigs. The iroquois homeobox 3 (*IRX3*) gene has been implicated in human obesity. Mouse experiments have shown that knocking out *IRX3* significantly enhances basal metabolism, reduces fat content, and controls body mass and composition. This study aimed to knock out *IRX3* using the CRISPR/Cas9 gene editing method to breed Bama minipigs with significantly reduced fat content. First, the CRISPR/Cas9 gene editing method was used to efficiently obtain *IRX3*^-/-^ cells. Then, the gene-edited cells were used as donor cells to produce surviving *IRX3*^-/-^ Bama minipigs using somatic cell cloning. The results show that the use of *IRX3*^-/-^ cells as donor cells for the production of somatic cell-cloned pigs results in a significant decrease in the average live litter size and a significant increase in the average number of stillbirths. Moreover, the birth weight of surviving *IRX3*^-/-^ somatic cell-cloned pigs is significantly lower, and viability is poor such that all piglets die shortly after birth. Therefore, the preliminary results of this study suggest that IRX3 may have important biological functions in pigs, and *IRX3* should not be used as a gene editing target to reduce fat content in Bama minipigs. Moreover, this study shows that knocking out *IRX3* does not favor the survival of pigs, and whether targeted regulation of *IRX3* in the treatment of human obesity will also induce severe adverse consequences requires further investigation.

## 1. Introduction

Bama minipigs are an excellent local pig breed unique to China. Their characteristics include small size, tolerance to rough feed, and strong adaptability, and disease resistance [1]. They have thin skin and are fleshy with a delicious taste, and thus, are utilized in local products with high economic value. Bama minipigs play an important role in poverty alleviation through industrial development at its place of origin. However, Bama minipigs also have significant defects such as high body fat content, low feed conversion rate, and slow growth speed, which limit their popularity and utilization.

The traditional genetic improvement of pigs generally involves long cycles and high costs, which, in turn, slows down the genetic breeding process for Bama minipigs. In recent years, the application of gene editing technology using clustered regularly interspaced short palindromic repeats (CRISPR)/CRISPR-associated (Cas) endonuclease 9 system (CRISPR/Cas9) in pigs has brought opportunities for the rapid improvement of economic traits of Bama pigs [2,3,4,5]. CRISPR/Cas9-mediated gene editing is a third-generation gene editing technology following the zinc finger endonuclease (ZFN) and transcription activator-like effector nuclease (TALEN) that allows gene knockout, insertion, and replacement at specific gene loci through homologous recombination (HR) and non-homologous end-joining (NHEJ), thereby achieving the purpose of site-specific modification [6]. The CRISPR/Cas9 gene editing technology is highly efficient and convenient for operation. Since its invention, it has been successfully used for gene editing in Drosophila [7], zebrafish [8], mouse [9,10], rat [11], rabbit [12,13], dog [14,15], pig [1,16,17,18,19,20,21,22], and cynomolgus monkey [23,24,25] and has demonstrated enormous advantages and potentials in animal disease models [26,27] and trait improvement [28,29]. Our research group had previously used CRISPR/Cas9 gene editing and somatic cell cloning technology to prepare gene-edited Bama minipigs [1], thereby laying a foundation for the improvement of productive traits of Bama minipigs by gene editing.

The production of transgenic cloned pigs with the aid of somatic cell nuclear transfer (SCNT), also named as somatic cell-cloning, is characterized by a disappointingly low efficiency. Therefore, significant improvement of pig cloning efficiency is required for practical applying SCNT strategies to various biomedical and biotechnological research fields [30,31,32,33,34,35]. Recent investigations have indicated a number of factors that bring about the generation of transgenic or non-transgenic cloned embryos, conceptuses and offspring in pigs. These factors encompass, among others: (1) the provenance of nuclear donor cells [36,37,38,39]; (2) nuclear, epigenomic and cytoplasmic maturity of enucleated metaphase II-stage oocytes [40,41,42,43]; (3) the methods used for artificially activating porcine SCNT-derived oocytes [44,45,46,47]; (4) capability of donor cell nuclear genome to be epigenetically reprogrammed in a cytoplasm of SCNT-derived oocytes and descendant blastomeres of cloned embryos [48,49,50,51,52,53]; (5) intergenomic communication between nuclear and mitochondrial DNA fractions in SCNT-derived oocytes and resultant cloned embryos [54,55,56,57]; and (6) initiation and progression of apoptosis processes in the ex vivo expanded nuclear donor cells and the cloned pig embryos generated [58,59,60,61,62,63,64].

In view of the high fat content of Bama minipigs, this study intended to target the obesity gene through CRISPR/Cas9 gene editing to reduce its fat content. To date, only a few functional genes directly related to porcine obesity have been reported. Therefore, we decided to identify relevant target genes that may be used for reference from known human obesity-associated genes. Iroquois homeobox 3 gene (*IRX3*) is a human obesity-related gene that has recently been identified [65,66]. Studies have shown that its up-regulation is significantly associated with human obesity, and mice with biallelic knockout mutations of *IRX3* knockout did not significantly reduce their viability after birth and can significantly enhance basal metabolism, reduce fat content, and ultimately reduce body weight, suggesting that *IRX3* is a major gene that controls body mass and composition in human and mouse [65,66]. However, the role of *IRX3* in pig has not been examined to date. This study knocked out bama pig *IRX3* using CRISPR/Cas9 gene editing technology to explore the relationship between *IRX3* and body weight and obesity in pig.

## 2. Materials and Methods

### 2.1. Animal Ethics

All the animal procedures used in this study were conducted in accordance with the Guide for Care and Use of Laboratory Animals (8th edition, released by the National Research Council, USA) and were approved by the Animal Care & Welfare Committee of Foshan University (approve no. 2019020). Pig ovaries used for producing in vitro maturated oocytes used as SCNT recipients were collected from a slaughterhouse. All animal surgical procedures were performed under anesthesia by a veterinarian, and all efforts were made to minimize animal suffering.

### 2.2. Reagents and Chemicals

Unless otherwise stated, all organic and inorganic reagents were purchased from Sigma-Aldrich Co. (St. Louis, MO, USA). Self-made solutions were filtered through a 0.22-μm filter (Millipore, Bedford, MA, USA) and stored at 4 °C or −20 °C until use. Pipette tips, centrifuge tubes, and petri dishes were purchased in aseptic packages and were all disposable.

### 2.3. Construction of CRISPR/Cas9 Plasmid

Design and construction of CRISPR/Cas9 plasmid were performed according to our previous study [1]. Two sgRNAs, namely sgRNA1 (CCCAGCTCGGATACCAGTACATC) and sgRNA2 (CCCCAGCTCGGATACCAGTACAT) (the protospacer adjacent motif (PAM) sequence was underlined), used for targeting exon 1 of *IRX3* (NCBI gene ID: 100518611) was designed by the CRISPR Design Tool (http://crispor.tefor.net). The pSpCas9(BB)-2A-Puro (PX459) V2.0 plasmids (Addgene #62988) were linearized by *Bsb*I restriction enzyme digestion, and linked with the annealed sgRNAs using a T4 DNA Ligase (TaKaRa, Dalian, China). The constructed CRISPR/Cas9 plasmid was confirmed by DNA sequencing (BGI, Shenzhen, China).

### 2.4. Cell Transfection and Selection of Gene-Edited Cell Colonies

Procedures used for the isolation, cultivation, and transfection of newborn Bama minipig kidney fibroblasts were based on our previous studies [1,41,67,68,69,70]. After establishing deep anesthesia, the piglet was euthanized and its bilateral kidneys were collected and minced in Dulbecco’s phosphate buffered saline (DPBS; Gibco, Grand Island, NY, USA). Tissue fragments were washed several times with DPBS and digested in 0.25% (w/v) trypsin-EDTA solution for 30 min at 37 °C. Isolated cells were cultured for 1 to 2 passages in Dulbecco’s modified Eagle’s medium (DMEM; Gibco) supplemented with 15% (v/v) fetal bovine serum (FBS; Gibco) and then stored frozen in liquid nitrogen.

Two days before transfection, the frozen cells at passages 1 to 2 were thawed and cultured in 4-well cell culture plates (NUNC, Shanghai, China) without antibiotics until cells reached approximately 50% confluency. The medium was replaced with fresh cell culture medium. Transfection mixtures were prepared with 1.5 µL Lipofectamine 3000 (Life Technologies, Carlsbad, CA, USA) plus 500 ng CRISPR/Cas9 plasmid, added to the cells, and then incubated overnight at 37 °C in a humidified atmosphere of 5% (v/v) CO_2_ in air. At 24 h post-transfection, cells were split into eight 6-well cell culture plates (NUNC). After 24 h of recovery, the transfected cells were selected with 0.75 µg/mL puromycin (Solarbio, Beijing, China) for 3 days. Then, puromycin was withdrawn and the cells were further cultured for 7 to 10 days. Individual cell colonies were picked out and cultured in 4-well cell culture plates. When confluency was achieved, the cell colonies were subcultured and a part of these was collected for genotyping. Positive gene-edited cell colonies were expanded and then cryopreserved.

### 2.5. Genotyping and Detection of Exogenous Genes Integration in Gene-Edited Cell Colonies

Genotyping of CRISPR/Cas9-mediated gene editing in pig cells was conducted according to our previous studies [1,70]. Genomic DNA was extracted from cell colonies and tail tissues of newborn cloned piglets using a TIANamp Genomic DNA Kit (Tiangen, Beijing, China). One pair of primers (PF: 5′-AGCAGATCAATAGGCGAACG-3′; PR2: 5′-TGTGACTGCGGAACCAAAAC-3′) were designed to amplify across the gene editing site, resulting in 617-bp amplicons. PCR reactions were conducted with 2 µL of genomic DNA, 0.5 µL forward primer (10 μM), 0.5 µL reverse primer (10 μM), 10 µL PrimeSTAR Max (TaKaRa, Dalian, China), and deionized water to a total volume of 20 µL. PCR amplification conditions were as follows: 1 cycle at 95 °C for 5 min; followed by 35 cycles at 98 °C for 10 s, 56 °C for 5 s, and 72 °C for 5 s; and then 72 °C for 5 min. The PCR products were examined by 1.5% (w/v) agarose gel electrophoresis containing 0.01% (v/v) Andy Gold™ Nucleic Acid Gel Stain (Applied BioProbes, Davis, CA, USA). The PCR amplicons were collected and ligated to an 18T vector (TaKaRa) for Sanger sequencing (BGI).

Detection of CRISPR/Cas9 plasmid integration in gene-edited cell colonies was conducted by PCR. One pair of specific primers (PF: 5′-CGGAGACTACAAGGATCATG-3′ and PR: 5′-TATCCTCTTCCACCAGGAAG-3′) was used for amplification of CRISPR/Cas9 plasmid, resulting in 428-bp amplicons. The glyceraldehyde-3-phosphate dehydrogenase (*GAPDH*) gene was used as reference using primers PF: 5′-TCTGCATCAGTGCTCCTTGA-3′ and PR: 5′-AAGAGGTGATGAAGCTCCGA-3′, resulting in 650-bp amplicons. Cell colonies integrated any of CRISPR/Cas9 plasmid were not used in generating somatic cell-cloned pigs.

### 2.6. Off-Target Detection in Gene-Edited Cell Colonies

Potential off-target sites (OTSs) were predicted by the CRISPR Design Tool (http://crispor.tefor.net) according to our previous studies [1,70]. Four sites with potential off-target effects (Table 1) were selected for detection of off-targets occurred in gene-edited cell colonies. Specific primers were used for PCR reaction, and the products were sequenced to confirm whether off-targeting mutations existed. Off-targets were identified by alignment of sequenced alleles to wild-type allele. Cell colonies harboring any off-targets were not used in generating somatic cell-cloned pigs.

### 2.7. Production of Gene-Edited Bama Minipigs by Somatic Cell Cloning

Procedures used for donor cell preparation, in vitro maturation of porcine oocytes, somatic cell nuclear transfer (SCNT), and embryo transfer were according to our previous studies [1,41,68,69]. Briefly, gene-edited cell colonies that did not have exogenous genes integrated and did not harbor any off-targets were used for producing mutant minipigs. To prepare nuclear transfer donor cells, cryopreserved mutant cells were thawed and cultured for 2 days, followed by serum starvation (DMEM supplemented with 0.5% FCS) for 48 h. The cells were then harvested and resuspended with 1 mL micromanipulation medium (10 mM HEPES-buffered TCM-199 containing 0.3% [w/v] bovine serum albumin [BSA]; pH = 7.3). This cell suspension was maintained at room temperature and used as SCNT donor cells. Cumulus-oocyte complexes (COCs) were aspirated from the follicles, and washed twice in PVA-TL-HEPES medium. The COCs were transferred into 200 µL drops of preheated maturation medium (bicarbonate-buffered TCM-199 supplemented with 0.1% [w/v] polyvinyl acetate [PVA], 3.05 mM D-glucose, 0.91 mM sodium pyruvate, 0.57 mM cysteine, 10 ng/mL epidermal growth factor [EGF], 0.5 µg/mL follicle-stimulating hormone [FSH], 0.5 µg/mL luteinizing hormone [LH], 0.0750 g/L penicillin G, 0.0500 g/L streptomycin and 10% [v/v] porcine follicular fluid), covered with mineral oil, and then incubated for 20 to 22 h at 38.5 °C in a humidified atmosphere of 5% (v/v) CO_2_ in air. Then, the COCs were cultured for an additional 20 h in the same medium without the gonadotropins. Following maturation, expanded cumulus cells were removed from the oocytes by vigorous pipetting in presence of 0.1% (w/v) hyaluronidase. Oocytes with an evenly granulated ooplasm and an extruded first polar body were selected and placed into the micromanipulation medium drop (containing donor cells and 7.5 µg/mL cytochalasin B) on a 60-mm cell culture dish (NUNC) and overlayed with mineral oil.

Matured oocytes were enucleated by aspirating the first polar body plus a portion of the adjacent cytoplasm (presumably containing the metaphase II plate) using a sharp-beveled glass pipette (WPI, Sarasota, FL, USA) with a diameter of 20 to 25 µm. After enucleation, a donor cell was carefully injected into the perivitelline space, maximizing the amount of cell membrane contact between the donor cell and the oocyte. The fusion and activation of nuclear transferred embryos were performed simultaneously using electrical pulses (2 successive DC pulses of 1.2 kV/cm for 30 μs; BTX2000, BTX Inc., San Diego, CA, USA) in a fusion medium [0.3 M mannitol, 1.0 mM CaCl_2_, 0.1 mM MgCl_2_, 0.5 mM HEPES plus 0.3% (w/v) BSA]. After fusion and activation, the reconstructed embryos were placed into PZM-3 containing 0.3% (w/v) BSA and cultured at 38.5 °C in a humidified atmosphere of 5% (v/v) CO_2_ in air. Fusion was checked 40 to 60 min later. Fused embryos were cultured until embryo transfer.

For embryo transfer, 200 to 300 cloned embryos were cultured 0 to 1 day *in vitro*, and then surgically transferred into the oviductal ampullary-isthmic junction of surrogate sows exhibiting natural estrus (within one day of the onset of estrus). Pregnancy was diagnosed by ultrasonography. Pregnant surrogate sows were delivered by natural parturition on days 114 to 120 of gestation (SCNT was performed on Day 0).

### 2.8. Identification of IRX3 Knockouts in Somatic Cell-Cloned Bama Minipigs

Tail biopsies were collected from newborn somatic cell-cloned piglets. One part of the tissue was used for extraction of genomic DNA, followed by PCR for identification of CRISPR/Cas9-mediated gene editing of the *IRX3* locus. The remaining tissue was used for extraction of total protein, which was used in western blot (WT) analysis for detecting the disruption of IRX3 protein expression. Briefly, tissue samples were homogenized in cell lysis buffer, and 30 µg of isolated total protein were analyzed by sodium dodecyl sulfate-polyacrylamide (SDS-PAGE) gel electrophoresis. Then, the distributed protein were immunoblotted onto a polyvinylidene fluoride membrane (Millipore Corp, MA, Bedford, USA). The primary mouse monoclonal antibodies against IRX3 (1:500; sc-166877, Santa Cruz) and GAPDH (1:500; sc-47724, Santa Cruz) were used and were detected with a horseradish peroxidase (HRP)-conjugated rabbit anti-mouse IgG secondary antibody (1:1000; sc-358914, Santa Cruz). The immuno-stained membranes were imaged using a Gel Doc EZ system (Bio-rad, Hercules, CA, USA) with immunochemiluminescent substrate (Tiangen) for detection of HRP. Positive WB results for the detection of IRX3 and GAPDH should appear at molecular weights of 60 and 38 kDa, respectively.

### 2.9. Statistical Analysis

Body weight and litter size data were expressed as the mean ± standard deviation (SD) and analyzed using independent sample, two-tailed student’s *t*-test. Statistical analysis was conducted with GraphPad Prism 5 software (La Jolla, CA, USA), and differences with a *p*-value < 0.05 were considered statistically significant.

## 3. Results

### 3.1. Design and Test of CRISPR/Cas9-Mediated IRX3 Gene Editing Target in Bama Minipig

**Figure 1:**
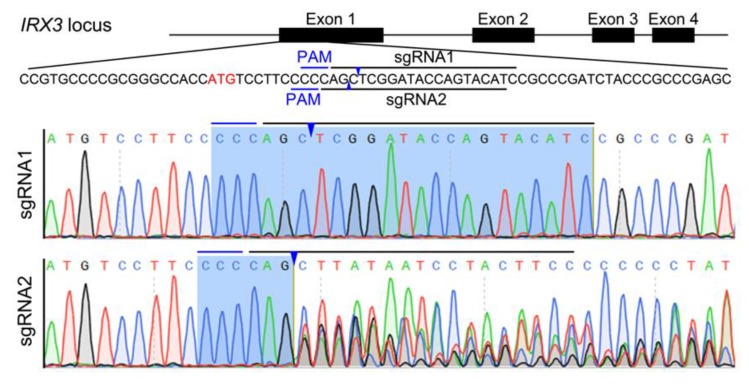
Design and assessment of sgRNAs targeting Bama minipig *IRX3*. Two potential sgRNAs (sgRNA1 and sgRNA2) were designed for targeting exon 1 of the Bama minipig *IRX3*. Two CRISPR/Cas9 vectors were individually introduced into cultured Bama minipig kidney fibroblast cells by Lipofectamine transfection to assess targeted DNA cleavage efficacy. The DNA sequencing results show that sgRNA2 caused a much higher proportion of targeted mutations than sgRNA1. The sgRNAs and protospacer adjacent motif (PAM) sequences are marked with the black and blue lines, respectively. The predicted cleavage sites are indicated by the blue arrowheads.

The Bama minipig *IRX3* gene has four exons, and exon 1 was used as the gene editing target in this study (Figure 1). Using the sgRNA online design tool (http://crispor.tefor.net), two targets with high scores were identified (recorded as sgRNA1 and sgRNA2, respectively) (Figure 1). CRISPR/Cas9 gene editing vectors that target sgRNA1 and sgRNA2 (referred to as Cas9-IRX3-sgRNA1 and Cas9-IRX3-sgRNA2, respectively) were constructed and tested in the kidney fibroblasts of Bama minipig. The two CRISPR/Cas9 vectors were separately transfected into Bama minipig kidney fibroblasts, the cells were harvested, and genomic DNA was extracted two days after transfection. The results of PCR and DNA sequencing showed that sgRNA2 caused a higher frequency of targeted mutations (Figure 1). Therefore, sgRNA2 was selected as the target to prepare *IRX3* gene-edited Bama minipig kidney fibroblasts.

### 3.2. Preparation of IRX3 Gene-Edited Bama Minipig Somatic Cells

Cas9-IRX3-sgRNA2, which is capable of efficient gene cleavage of the target sequence of *IRX3* in Bama minipig somatic cells, was used for the preparation of gene-edited cells. The plasmid was transfected into Bama minipig kidney fibroblasts using a liposome transfection method. After puromycin resistance screening and continuous culture, a total of eight single-cell colonies with good morphology were selected and expanded in culture. A fraction of the cells was used for genetic identification, and the remaining cells were frozen for later use.

The *IRX3* target sequence of the transfected cells was obtained by PCR and ligated to an 18T vector for Sanger sequencing. The results showed that seven out of eight single cell colonies (87.5%) had mutations on both alleles (Table 2). After analyzing the results of gene mutations, we found that the two *IRX3* alleles of single-cell colony #2-2 had frameshift mutations due to a deletion and insertion of one base, respectively. In view of its minimal genetic changes and good cell morphology and viability, we selected this single-cell colony for subsequent analysis.

**Figure 2:**
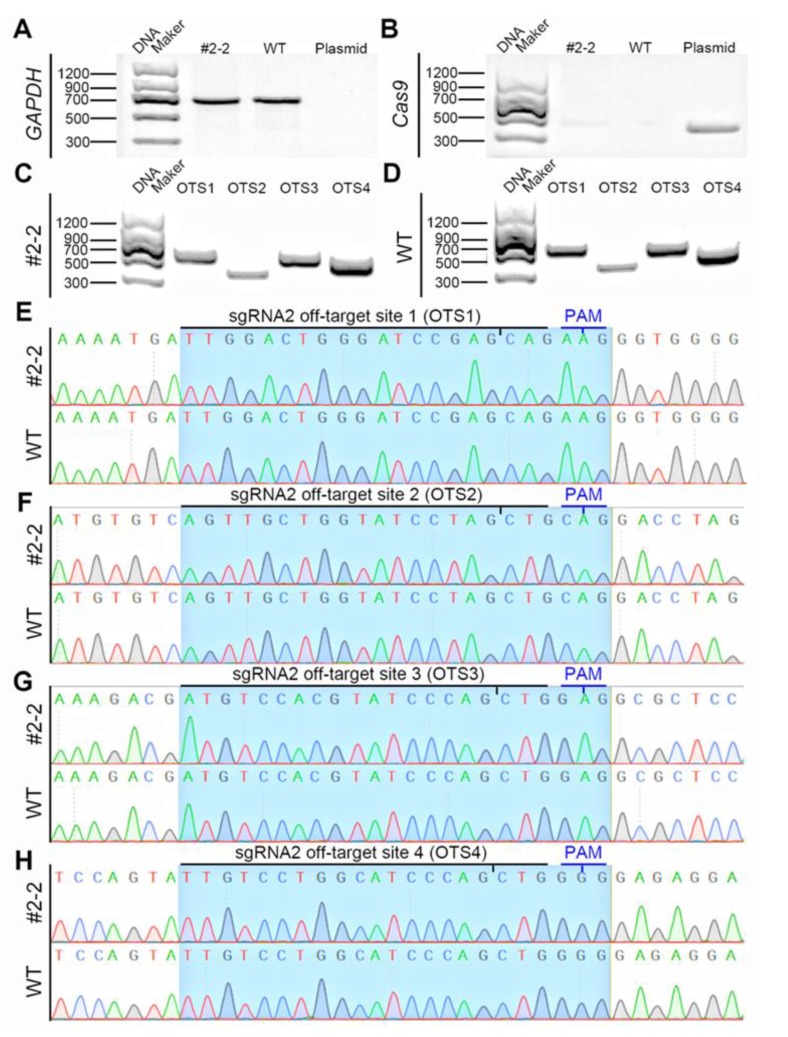
Detection of CRISPR/CAS9 plasmid integration and off-target in gene-edited single-cell colony. PCR amplification confirmed no integration of CRISPR/Cas9 plasmid in single-cell colony #2-2 (**A**). PCR amplification of glyceraldehyde-3-phosphate dehydrogenase gene (*GAPDH*) was used to confirm the presence of genomic DNA in all the samples (**B**). Four sites with potential off-target effects (OTS) were selected for detection of off-targets occurred in gene-edited cell colonies. DNA sequencing results showed no genetic mutations in these four OTS regions in single-cell colony #2-2 (**C**–**H**). The sample collected from a wild-type (WT) Bama minipig was used as a control.

The integration of foreign gene editing plasmids into the cell genome affects the phenotypic analysis of gene-edited animals. Therefore, we performed PCR amplification of single-cell colony #2-2 to detect the presence of foreign gene integration. PCR analysis showed no integration of CRISPR/Cas9 plasmid into single-cell colony #2-2 (Figure 2A,B).

CRISPR/Cas9 editing of the genome of a cell may also result in off-target effects. We selected four highly active potential OTSs from the off-target prediction provided by the sgRNA online design tool (http://tools.genome-engineering.org) for testing (Table 1). DNA sequencing results showed no genetic mutations in these four OTS regions in single-cell colony #2-2 (Figure 2C–F).

In summary, using the CRISPR/Cas9-mediated gene editing method, we obtained single-cell colony #2-2 that carried biallelic frameshift mutations of *IRX3* and confirmed that no foreign gene integration and off-target effects occurred, and the cells demonstrated good morphology and viability. Therefore, these cells were used for the production of cloned pigs from gene-edited somatic cells.

### 3.3. Biallelic Knockout of IRX3 Significantly Reduces the Production and Survival of Somatic Cell-Cloned Bama Minipigs

**Figure 3:**
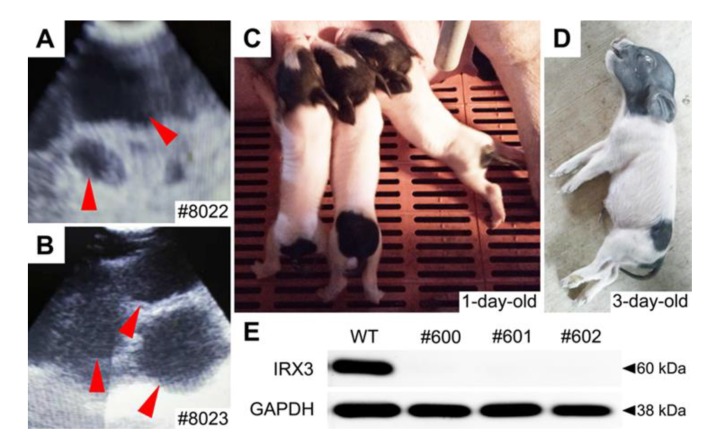
Production of *IRX3* knockout Bama minipigs. Two surrogate sows became pregnant, which was identified using B-ultrasound scanning (**A**,**B**; the red arrows indicate the gestational sacs), and successfully gave birth to three alive piglets (**C**). The Bama minipigs were in poor condition after birth and had difficulty with feeding that even after careful care, all died within three days after birth (panel (**D**) shows one of them). Western blotting revealed that all of the gene-edited Bama minipigs did not express the IRX3 protein, indicating that a complete knockout of IRX3 was achieved (**E**). Glyceraldehyde-3-phosphate dehydrogenase (GAPDH) was used as reference. The sample collected from a wild-type (WT) piglet was used as control.

Single-cell colony #2-2 cells were used as donor cells for the production of cloned pigs. A total of 1,000 cloned embryos were transplanted into four surrogate sows (Table 3), of which two were pregnant (Figure 3A,B) and three live cloned pigs were later born (Figure 3C), indicating an overall cloning efficiency of 0.3%.

**Figure 4:**
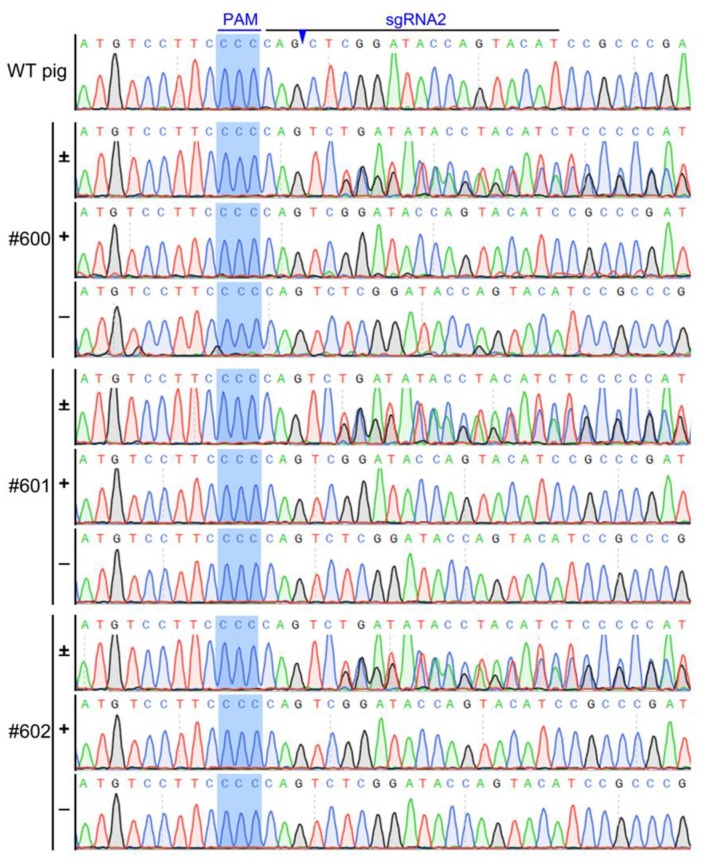
Genotyping of *IRX3* gene-edited Bama minipigs. The gene sequencing results show that all three live-birth somatic cell-cloned Bama minipigs (#600, #601 and #602) harbored biallelic frameshift mutations in CRISPR/CAS9 targeted *IRX3* locus due to a deletion and insertion of one base, respectively. Symbols as “+” and “−” respectively show the DNA sequences from single allele ligated to an 18T vector for Sanger sequencing. Symbols “±” show the DNA sequences from pooled PCR amplicons. The sample collected from a wild-type (WT) piglet was used as control. The sgRNA2 and protospacer adjacent motif (PAM) sequences are marked with the black and blue lines, respectively. The predicted cleavage sites are indicated by the blue arrowheads.

Tail tissues of cloned piglets were collected for genotyping and protein expression analysis. Gene sequencing results showed that the genotype of the cloned Bama minipigs were the same as that of single-cell colony #2-2, all of which harbored biallelic frameshift mutations (Figure 4). Western blotting revealed that all of the gene-edited Bama minipigs did not express the IRX3 protein indicating that a complete knockout of *IRX3* was achieved (Figure 3E).

**Figure 5:**
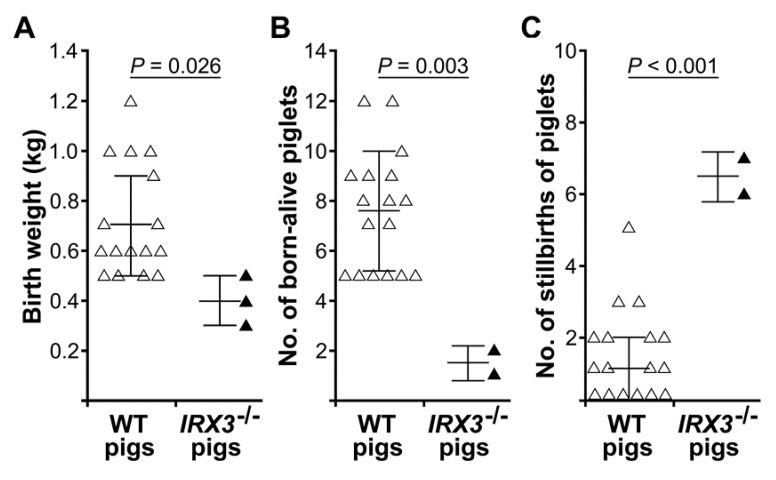
Biallelic *IRX3* knockouts significantly reduces the production and survival of somatic cell-cloned Bama minipigs. The *IRX3^-/-^* Bama minipigs at birth were abnormally small and weak, with a birth weight significantly lower than the weight of non-gene-edited somatic cell-cloned Bama minipigs produced during the same period (*p* < 0.05) (**A**). In addition, compared with the production of non-gene-edited somatic cell-cloned Bama minipigs that were performed during the same period, using *IRX3^-/-^* donor cells significantly affected the production of somatic cell-cloned Bama minipigs, with a significant decrease in average live litter size (*p* < 0.01) (**B**), and a significant increase in average litter stillbirths (*p* < 0.01) (**C**). Data were expressed as the mean ± standard deviation (SD) and analyzed with independent sample, two-tailed student’s *t*-test. Differences with *p*-values < 0.05 were considered statistically significant.

The *IRX3^-/-^* Bama minipigs at birth were abnormally small and weak, with birth weights of roughly 0.4 ± 0.1 kg, which was significantly lower than the weight of non-gene-edited somatic cell-cloned Bama minipigs produced during the same period (0.7 ± 0.2 kg; *p* < 0.05) (Figure 5A). In addition, the *IRX3^-/-^* Bama minipigs were in poor condition after birth and had difficulty with feeding that even after careful care, all died within three days after birth (Figure 3D).

Retrospective analysis of the production data of the somatic cell-cloned pigs showed that compared with the production of non-gene-edited somatic cell-cloned Bama minipigs that were performed during the same period and using the same production method and using the same production method, using *IRX3^-/-^* donor cells significantly affected the production of somatic cell-cloned Bama minipigs, with a significant decrease in average live litter size (7.6 ± 2.4 vs. 1.5 ± 0.7, *p* < 0.01) (Figure 5B), and a significant increase in average litter stillbirths (1.4 ± 1.4 vs. 6.5 ± 0.7, *p* < 0.01) (Figure 5C).

Considering that off-target effects had been ruled out in the donor cells used to produce the *IRX3^-/-^* Bama minipigs, the results of this study suggest that *IRX3* might have important biological functions in pigs, and that biallelic knockout mutations in *IRX3* significantly affect the production and survival of somatic cell-cloned pigs. Therefore, the result of this study indicates that *IRX3* is probably not a suitable gene editing target for reducing fat content in Bama minipigs.

## 4. Discussion

To date, traditional genetic breeding remains the main means of improving the economic traits of livestock. The emergence of gene editing technologies as represented by CRISPR/Cas9 has provided accurate and efficient genetic improvement methods for animals and plants, including pigs. Currently, CRISPR/Cas9 gene editing technology has been successful in pigs. For example, in 2017, Burkard et al. [20] used CRISPR/Cas9 technology to knock out *CD163*, a cell receptor involved in the invasion of porcine reproductive and respiratory syndrome virus, and successfully obtained gene-edited pigs capable of resisting the infection of reproductive and respiratory syndrome virus. Moreover, Xie et al. [21] used CRISPR/Cas9-mediated gene knock-in technology to successfully obtain gene-edited pigs that express interference RNA resisting the classic swine fever virus, and in vivo and in vitro virus-challenging experiments confirmed that the gene-edited pigs could effectively resist swine fever virus infection, which thereby significantly reduced the clinical symptoms and pig death caused by swine fever virus. These cases indicate that the CRISPR/Cas9 gene editing technology has an important application value in the genetic improvement of pigs.

Historically, China’s pig breeding program has focused on genetic selection towards reducing fat deposition and increasing lean meat content. In the past a few decades, genes related to economic traits such as promoting growth, muscle formation, and fat consumption have been identified, and these economic traits can be improved through gene editing. For example, insulin-like growth factor 2 (IGF2) plays an important role in cell proliferation and differentiation [71]. Using CRISPR/Cas9 gene editing to upregulate *IGF2* gene expression can accelerate the growth of pigs [29,72]. Myostatin (MSTN) plays an important negative regulatory role in the proliferation, differentiation, and growth of animal skeletal muscles. MSTN gene knockout or reduced expression of MSTN will promote pig muscle growth and increase lean meat proportion [16,18]. Uncoupling protein 1 (UCP1) plays an important role in energy conversion and maintaining body temperature. The lack of functional UCP1 in pigs thus results in an increase in fat content. Using CRISPR/Cas9 gene editing to restore *UCP1* gene activity in pigs can promote the consumption of energy materials and fat conversion, which increase lean meat proportion and at the same time improve cold resistance of pigs [28]. The aforementioned cases all resulted in reduced fat content in pigs by promoting muscle growth or accelerating fat conversion. To date, no study directly targeting lipogenesis in pig through gene editing has been conducted.

IRX3 is closely related to fat formation and metabolism and ultimately affects body weight. The elucidation of *IRX3* function stems from research on human obesity [65,66]. *FTO* is the first human obese gene identified, while subsequent research has shown that resistance to obesity does not come from *FTO*. Smemo et al. [66] first revealed that FTO regulates the expression of the *IRX3* gene, and the classic obesity risk site rs9930506 of *FTO* does not change the mRNA level of the *FTO* gene in brain tissues, but is associated with changes in *IRX3* expression. In mouse and zebrafish embryos, adult mouse brain, and human cells, the first intron of *FTO* is close to the adjacent IRX3 gene in chromosome conformation and functions as an enhancer to regulate *IRX3* expression [66]. Claussnitzer et al. [65] identified the presence of an enhancer-acting element for *IRX3* within a 10-kb gene range of the first intron of *FTO* in human cells, which contains a highly conserved variant of rs1421085 in which wild-type allele is T, whereas mutated allele with obesity risk is C. Under normal circumstance (wild-type: T), the *cis*-acting element, AATArITll, binds to the transcription factor ARID5B to inhibit the expression of *IRX3*, thereby promoting the expression of *UCP1* in white fat tissues, increasing the basal metabolic rate, and inhibiting the occurrence of obesity [65]. If the *cis*-acting element is changed from T to C, AACATF (rsl421085) cannot bind to ARID5B, and no inhibitory effect of ARID5B is elicited, leading to the upregulation of *IRX3* by more than two-fold, thereby inhibiting the expression of *UCP1*, reducing basal metabolic rate, and promoting the onset of obesity. Knocking out *IRX3* in mouse adipose tissues increases browning of adipose tissues, enhances basal metabolic rate, and reduces body weight by 25–30%, suggesting that *IRX3* is an important gene that directly controls body weight and is considered to be a potential target for the treatment of human obesity [66].

Although Bama minipigs have good developmental values, their fat content is too high. A moderately high intramuscular fat content can improve the taste and flavor of pork, while an excessively high fat content is not conducive to human health and affects its food value. High fat content also directly leads to low feed conversion rate, which results in an increase in feeding costs. This study intended to explore whether *IRX3* gene knockout could reduce fat content in pig using the CRISPR/Cas9 gene editing method to knockout *IRX3* in Bama minipigs. In this study, CRISPR/Cas9-mediated gene editing was used to obtain somatic cells with a biallelic frameshift mutation of *IRX3*, and foreign gene integration and off-target effects were excluded. Subsequently, live somatic cell-cloned Bama minipigs were successfully generated, and biallelic knockout mutations in *IRX3* were achieved at the genome and protein levels, as confirmed by gene sequencing and western blotting, respectively. At birth, the *IRX3^-/-^* Bama minipigs were abnormally thin and weak, with body weights significantly lower than the control group. In addition, they demonstrated poor survivability such as feeding difficulties, and all died three days after birth despite providing extensive care. The production data of somatic cell-cloned pigs also showed that compared with the production of non-gene-edited somatic cell-cloned Bama minipigs, the use of *IRX3^-/-^* donor cells significantly affected the production of somatic cell-cloned pigs, with specific manifestations of significantly lower average live litter size and significantly higher average litter stillbirths. This is different from previous studies in mouse that biallelic knockout mutations in *IRX3* did not significantly reduce the viability of the mice after birth [65]. This may be due to species differences in that IRX3 may have other important biological functions in pig, so that biallelic knockout of *IRX3* directly affects pig health.

Previous studies have shown that IRX3 plays a key role in regulating intercellular junctions and cardiac electrophysiological transmission [73]. Under normal heart conditions, the left and the right ventricles are simultaneously electrically activated. Mice with *IRX3* knocked out exhibit abnormal cardiac electrophysiology, including extended QRS complex duration, extended conduction time between the atrioventricular bundle and the ventricles, and right bundle branch block (RBBB) [74]. The heart is the driving force of blood circulation throughout the entire body. When heart function is inadequate, the pumping capacity is reduced, and adequate tissue perfusion pressure cannot be maintained. Thus, oxygen and nutrients cannot effectively reach the tissues to participate in metabolism, and at the same time, the waste generated during tissue metabolism are not eliminated immediately, which can lead to dysfunction of the body and endanger life. The generation of the *IRX3* gene-deficient pigs to comprehensively explore the functional mechanism of IRX3 in pigs, particularly in relation to cardiac function, is warranted.

In summary, this study successfully generated an *IRX3^-/-^* Bama minipig model using CRISPR/Cas9 gene editing and somatic cell cloning technology and observed that *IRX3^-/-^* donor cells lead to a significant decrease in the production of somatic cell-cloned pigs, which was specifically manifested as a significant reduction in the average live litter size, a marked increase in the average number of litter stillbirths, significant reduction in birth weight of surviving *IRX3^-/-^* somatic cell-cloned pigs, and poor viability after birth. Therefore, the preliminary results of this study suggest that *IRX3* may have important biological functions in pig, and *IRX3* should not be used as a gene editing target to reduce fat content in Bama minipigs. Moreover, because this study shows that complete knockout of *IRX3* is lethal to pigs, whether targeted regulation of *IRX3* for the purpose of treating human obesity also causes severe adverse consequences requires further investigation.

## Figures and Tables

**Table 1:** Off-target sites (OTS) and corresponding primers used for analysis of off-targets.

OTS No.	Chr.	Strand	Position	Sequence *	Score	Gene	Primers for PCR and Sequencing	Amplicon (bp)
sgRNA2	Chr6	−1	31046354	ATGTACTGGTATCCGAGCTG**GGG**	100	NCBI Gene ID: 100518611	PF: AGCAGATCAATAGGCGAACG PR: CTGTCCTTCAGCTCATACTG	617
OTS1	Chr13	1	116315529	TTGGACTGGGATCCGAGCAG**AAG**	0.49	NCBI Gene ID: 106505748	PF: TCAACTTCTCGCATGGTGTG PR: TGACTAGCAACTTCAGAGGC	505
OTS2	Chr14	−1	34640565	AGTTGCTGGTATCCTAGCTG**CAG**	0.40	NCBI Gene ID: 100153965	PF: TCTCCAGGTATTATCAGGAGT PR: CACATCCTATAAAGCTCAGTC	347
OTS3	Chr12	1	53174151	ATGTCCACGTATCCCAGCTG**GAG**	0.27	NCBI Gene ID: 100523637	PF: AAGTCGGAGAGGTTGGTATC PR: TTCCTACAGAGCAGAAACCG	500
OTS4	Chr17	1	56522870	TTGTCCTGGCATCCCAGCTG**GGG**	0.38	None	PF: AACCTAGAGCTGTGGACAAC PR: CTCCAACACTTGTAGCCTTG	451

* Underlined letters are the mismatched nucleotides in off-target sequences aligned to sgRNA. PAM sequences are marked in bold.

**Table 2:** Summary of *IRX3* genotypes of single-cell colonies generated by CRISPR/Cas9-mediated gene editing.

Colony No.	Allele	*IRX3* Genotypes *	Indels
Wild type	1/2	ATGTCCTTCC**CCC**AGCTCGGATACCAGTACATCCGCCCGATCTACCCGCCCGAGC	WT/WT
#2-1	1	ATGTCCTTCC**CCC**AG------------TACATCCGCCCGATCTACCCGCCCGAGC	-12
	2	ATGTCCTTCC**CCC**AGCCTCGGATACCAGTACATCCGCCCGATCTACCCGCCCGAG	+1
#2-2	1	ATGTCCTTCC**CCC**AG-TCGGATACCAGTACATCCGCCCGATCTACCCGCCCGAGC	-1
	2	ATGTCCTTCC**CCC**AGTCTCGGATACCAGTACATCCGCCCGATCTACCCGCCCGAG	+1
#2-3	1/2	ATGTCCTTCC**CCC**AG------------TACATCCGCCCGATCTACCCGCCCGAGC	-12/-12
#2-4	1/2	ATGTCCTTCC**CCC**AG------------TACATCCGCCCGATCTACCCGCCCGAGC	-12/-12
#2-5	1/2	ATGTCCTTCC**CCC**AGCTCGGATACCAGTACATCCGCCCGATCTACCCGCCCGAGC	WT/WT
#2-6	1	ATGTCCTTCC------TCGGATACCAGTACATCCGCCCGATCTACCCGCCCGAGC	-6
	2	ATGTCCTTCC---------GATACCAGTACATCCGCCCGATCTACCCGCCCGAGC	-9
#2-7	1/2	ATG-------------TCGGATACCAGTACATCCGCCCGATCTACCCGCCCGAGC	-13/-13
#2-8	1	ATGTCCTTCC**CCC**AG--CGGATACCAGTACATCCGCCCGATCTACCCGCCCGAGC	-2
	2	ATGTCCTTCC**CCC**AG-----ATACCAGTACATCCGCCCGATCTACCCGCCCGAGC	-5

Notes: * The sgRNA2 sequences are underlined, protospacer adjacent motif (PAM) sequences are labeled with bold fonts. Deletions in the targeted sequences are replaced with “-”.

**Table 3:** Production of gene-edited and un-gene-edited somatic cell-cloned Bama minipigs.

Recipient Sow	Donor Cells	Gender	No. of Embryos Transferred	Day 40 Pregnancy Status *	Gestational Period(day)	No. of Piglets Delivered(Alive/Stillborn)	Cloning Efficiency(%) **	Birth Weight(Kg)
#8024	IRX^-/-^	♂	250	-				
#8025	IRX^-/-^	♂	250	-				
#8022	IRX^-/-^	♂	250	+	117	1/6		
#8023	IRX^-/-^	♂	250	+	116	2/7		
Sum		♂	1000			3/13	0.3%	0.3; 0.4; 0.5
#1757	WT	♂	280	+	N/A	9/0		0.6; 1.0; 0.6; 0.6; 0.7; 1.0; 0.5; 0.6; 1.2
#0205	WT	♂	230	+	N/A	7/1		1.0; 0.5; 0.6; 0.9; 0.7; 0.5; 0.5
#4993	WT	♂	230	+	N/A	5/2		N/A
#0207	WT	♂	212	+	N/A	8/0		N/A
#2020	WT	♂	246	+	N/A	10/2		N/A
#4998	WT	♂	243	+	N/A	12/1		N/A
#4990	WT	♂	240	+	N/A	12/0		N/A
#2021	WT	♂	230	+	N/A	9/0		N/A
#0706	WT	♂	230	+	N/A	8/2		N/A
#7388	WT	♂	235	+	N/A	7/1		N/A
#1801	WT	♂	220	+	N/A	5/0		N/A
#1123	WT	♂	220	+	N/A	9/0		N/A
#1215	WT	♂	220	+	N/A	5/5		N/A
#1802	WT	♂	220	+	N/A	5/3		N/A
#0207	WT	♂	280	+	N/A	8/0		N/A
#0208	WT	♂	280	+	N/A	5/3		N/A
#0307	WT	♂	280	+	N/A	5/2		N/A
Sum			4096			129/23	N/A	

Notes: * Pregnancy status: +, pregnant; -, not pregnant; ** Cloning efficiency: No. of live piglets born / No. of embryos transferred × 100%. WT, wild type, means non-gene-edited somatic cells. WT and IRX^-/-^ cells are from the same donor animal origin. N/A, Not Applicable.

## References

[1] Zhu X.X., Zhong Y.Z., Ge Y.W., Lu K.H., Lu S.S. (2018). CRISPR/Cas9-mediated generation of Guangxi Bama minipigs harboring three mutations in α-synuclein causing Parkinson’s disease. Sci. Rep..

[2] Ruan J.X., Xu J., Chen-Tsai R.Y., Li K. (2017). Genome editing in livestock: Are we ready for a revolution in animal breeding industry?. Transgenic Res..

[3] Peterson B. (2017). Basics of genome editing technology and its application in livestock species. Reprod Domest Anim..

[4] Ryu J.H., Prather R.S., Lee K. (2018). Use of gene-editing technology to introduce targeted modifications in pigs. J. Anim. Sci. Biotechno..

[5] Zhao J.G., Lai L.X., Ji W.Z., Zhou Q. (2019). Genome editing in large animals: Current status and future prospects. Natl. Sci. Rev..

[6] Hsu P.D., Lander E.S., Zhang F. (2014). Development and applications of CRISPR-Cas9 for genome engineering. Cell.

[7] Bassett A.R., Tibbit C., Ponting C.P., Liu J.L. (2013). Highly efficient targeted mutagenesis of Drosophila with the CRISPR/Cas9 system. Cell Rep..

[8] Hwang W.Y., Fu Y.F., Reyon D., Maeder M.L., Tsai S.Q., Sander J.D., Peterson R.T., Yeh J.R.J., Joung J.K. (2013). Efficient genome editing in zebrafish using a CRISPR-Cas system. Nat. Biotechnol..

[9] Wang H., Yang H., Shivalila C.S., Dawlaty M.M., Cheng A.W., Zhang F., Jaenisch R. (2013). One-step generation of mice carrying mutations in multiple genes by CRISPR/Cas-mediated genome engineering. Cell.

[10] Yang H., Wang H., Shivalila C.S., Cheng A.W., Shi L., Jaenisch R. (2013). One-step generation of mice carrying reporter and conditional alleles by CRISPR/Cas-mediated genome engineering. Cell.

[11] Li W., Teng F., Li T., Zhou Q. (2013). Simultaneous generation and germline transmission of multiple gene mutations in rat using CRISPR-Cas systems. Nat. Biotechnol..

[12] Lv Q.Y., Yuan L., Deng J.C., Chen M., Wang Y., Zeng J., Li Z.J., Lai L.X. (2016). Efficient generation of myostatin gene mutated rabbit by CRISPR/Cas9. Sci. Rep..

[13] Sui T., Lau Y.S., Liu D., Liu T., Xu L., Gao Y., Lai L., Li Z., Han R. (2018). A novel rabbit model of Duchenne muscular dystrophy generated by CRISPR/Cas9. Dis. Mod. Mech..

[14] Zou Q.J., Wang X.M., Liu Y.Z., Ouyang Z., Long H.B., Wei S., Xin J., Zhao B.T., Lai S., Shen J. (2015). Generation of gene-target dogs using CRISPR/Cas9 system. Mol. Cell Biol..

[15] Feng C., Wang X.M., Shi H., Yan Q.M., Zheng M., Li J., Zhang Q.J., Qin Y.M., Zhong Y.G., Mi J. (2018). Generation of ApoE deficient dogs via combination of embryo injection of CRISPR/Cas9 with somatic cell nuclear transfer. J. Genet. Genom..

[16] Wang K.K., Ouyang H.S., Xie Z.C., Yao C.G., Guo N.N., Li M.J., Jiao H.P., Pang D.X. (2015). Efficient generation of myostatin mutations in pigs using the CRISPR/Cas9 system. Sci. Rep..

[17] Wang X.L., Cao C.W., Huang J.J., Yao J., Hai T., Zheng Q.T., Wang X., Zhang H.Y., Qin G.S., Cheng J.B. (2016). One-step generation of triple gene targeted pigs using CRISPR/Cas9 system. Sci. Rep..

[18] Bi Y.Z., Hua Z.D., Liu X.M., Hua W.J., Ren H.Y., Xiao H.W., Zhang L.P., Li L., Wang Z.R., Laible G. (2016). Isozygous and selectable marker-free MSTN knockout cloned pigs generated by the combined use of CRISPR/Cas9 and Cre/LoxP. Sci. Rep..

[19] Whitworth K.M., Rowland R.R.R., Ewen C.L., Trible B.R., Kerrigan M.A., Cino-Ozuna A.G., Samuel M.S., Lightner J.E., Mclaren D.G., Mileham A.J. (2016). Gene-edited pigs are protected from porcine reproductive and respiratory syndrome virus. Nat. Biotechnol..

[20] Burkard C., Lillico S.G., Reid E., Jackson B., Mileham A.J., Ait-Ali T., Whitelaw C.B.A., Archibald A.L. (2017). Precision engineering for PRRSV resistance in pigs: Macrophages from genome edited pigs lacking CD163 SRCR5 domain are fully resistant to both PRRSV genotypes while maintaining biological function. PLoS Pathog..

[21] Xie Z.C., Pang D.X., Yuan H.M., Jiao H.P., Lu C., Wang K.K., Yang Q.B., Li M.J., Chen X., Yu T.T. (2018). Genetically modified pigs are protected from classical swine fever virus. PLoS Pathog..

[22] Niu D., Wei H.J., Lin L., George H., Wang T., Lee I.H., Zhao H.Y., Wang Y., Kan Y.N., Shrock E. (2017). Inactivation of porcine endogenous retrovirus in pigs using CRISPR/Cas9. Science.

[23] Niu Y.Y., Shen B., Cui Y.Q., Chen Y.C., Wang J.Y., Wang L., Kang Y., Zhao X.Y., Si W., Li W. (2014). modified cynomolgus Generation of gene- monkey via Cas9/RNA-mediated gene targeting in one-cell embryos. Cell.

[24] Yao X., Liu Z., Wang X., Wang Y., Nie Y.H., Lai L., Sun R.L., Shi L.Y., Sun Q., Yang H. (2018). Generation of knock-in cynomolgus monkey via CRISPR/Cas9 editing. Cell Res..

[25] Zuo E.W., Cai Y.J., Li K., Wei Y., Wang B.A., Sun Y.D., Liu Z., Liu J.W., Hu X.D., Wei W. (2017). One-step generation of complete gene knockout mice and monkeys by CRISPR/Cas9-mediated gene editing with multiple sgRNAs. Cell Res..

[26] Yan S., Tu Z.C., Liu Z.M., Fan N.N., Yang H.M., Yang S., Yang W.L., Zhao Y., Ouyang Z., Lai C.D. (2018). A huntingtin knockin pig model rapitulates features of selective neurodegeneration in Huntington’s disease. Cell.

[27] Fang B., Ren X.Y., Wang Y., Li Z., Zhao L.H., Zhang M.L., Li C., Zhang Z.W., Chen L., Li X.X. (2018). Apolipoprotein E deficiency accelerates atherosclerosis development in miniature pigs. Dis. Model. Mech..

[28] Zheng Q.T., Lin J., Huang J.J., Zhang H.Y., Zhang R., Zhang X.Y., Cao C.W., Hambly C., Qin G.S., Yao J. (2017). Reconstitution of UCP1 using CRISPR/Cas9 in the white adipose tissue of pigs decreases fat deposition and improves thermogenic capacity. Proc. Natl. Acad. Sci. USA.

[29] Xiang G.H., Ren J.L., Hai T., Fu R., Yu D.W., Wang J., Li W., Wang H.Y., Zhou Q. (2018). Editing porcine IGF2 regulatory element improved meat production in Chinese Bama pigs. Cell Mol. Life Sci..

[30] Claussnitzer M., Dankel S.N., Kim K.H., Quon G., Meuleman W., Haugen C., Glunk V., Sousa L.S., Beaudry J.L., Puviindran V. (2015). FTO obesity variant circuitry and adipocyte browning in humans. New Engl. J. Med..

[31] Lee S.C., Lee H., Oh K.B., Hwang I.S., Yang H., Park M.R., Ock S.A., Woo J.S., Im G.S., Hwang S. (2017). Production and breeding of transgenic cloned pigs expressing human CD73. Dev. Reprod..

[32] Samiec M. (2004). Development of pig cloning studies: Past, present and future. J Anim. Feed Sci..

[33] Callesen M.M., Árnadóttir S.S., Lyskjaer I., Ørntoft M.W., Høyer S., Dagnaes-Hansen F., Liu Y., Li R., Callesen H., Rasmussen M.H. (2017). A genetically inducible porcine model of intestinal cancer. Mol Oncol..

[34] Samiec M., Skrzyszowska M. (2011). Transgenic mammalian species, generated by somatic cell cloning, in biomedicine, biopharmaceutical industry and human nutrition/dietetics–recent achievements. Pol. J. Vet. Sci..

[35] Samiec M., Skrzyszowska M. (2011). The possibilities of practical application of transgenic mammalian species generated by somatic cell cloning in pharmacology, veterinary medicine and xenotransplantology. Pol. J. Vet. Sci..

[36] Kim G.A., Lee E.M., Cho B., Alam Z., Kim S.J., Lee S., Oh H.J., Hwang J.I., Ahn C., Lee B.C. (2019). Generation by somatic cell nuclear transfer of GGTA1 knockout pigs expressing soluble human TNFRI-Fc and human HO-1. Transgenic Res..

[37] Lee J., Lee Y., Lee G.S., Lee S.T., Lee E. (2019). Comparative study of the developmental competence of cloned pig embryos derived from spermatogonial stem cells and fetal fibroblasts. Reprod Domest Anim..

[38] Opiela J., Samiec M., Romanek J. (2017). In vitro development and cytological quality of inter-species (porcine bovine) cloned embryos are affected by trichostatin A-dependent epigenomic modulation of adult mesenchymal stem cells. Theriogenology.

[39] Li Z., He X., Chen L., Shi J., Zhou R., Xu W., Liu D., Wu Z. (2013). Bone marrow mesenchymal stem cells are an attractive donor cell type for production of cloned pigs as well as genetically modified cloned pigs by somatic cell nuclear transfer. Cell Reprogram..

[40] Samiec M., Skrzyszowska M. (2010). Preimplantation developmental capability of cloned pig embryos derived from different types of nuclear donor somatic cells. Ann Anim Sci..

[41] Wang X.F., Zhu X.X., Liang X.W., Xu H.Y., Liao Y.Y., Lu K.H., Lu S.S. (2019). Effects of resveratrol on in vitro maturation of porcine oocytes and subsequent early embryonic development following somatic cell nuclear transfer. Reprod Domest Anim..

[42] Wang H., Cui W., Meng C., Zhang J., Li Y., Qian Y., Xing G., Zhao D., Cao S. (2018). MC1568 enhances histone acetylation during oocyte meiosis and improves development of somatic cell nuclear transfer embryos in pig. Cell Reprogram..

[43] Samiec M., Skrzyszowska M. (2012). High developmental capability of porcine cloned embryos following trichostatin A-dependent epigenomic transformation during in vitro maturation of oocytes pre-exposed to R-roscovitine. Anim. Sci. Pap. Rep..

[44] Gupta M.K., Heo Y.T., Kim D.K., Lee H.T., Uhm S.J. (2019). 5-Azacytidine improves the meiotic maturation and subsequent in vitro development of pig oocytes. Anim. Reprod Sci..

[45] Samiec M., Skrzyszowska M. (2014). Biological transcomplementary activation as a novel and effective strategy applied to the generation of porcine somatic cell cloned embryos. Reprod Biol..

[46] Samiec M., Skrzyszowska M. (2010). The use of different methods of oocyte activation for generation of porcine fibroblast cell nuclear-transferred embryos. Ann. Anim. Sci..

[47] Bang J.I., Yoo J.G., Park M.R., Shin T.S., Cho B.W., Lee H.G., Kim B.W., Kang T.Y., Kong I.K., Kim J.H. (2013). The effects of artificial activation timing on the development of SCNT-derived embryos and newborn piglets. Reprod Biol..

[48] Samiec M., Skrzyszowska M. (2012). Roscovitine is a novel agent that can be used for the activation of porcine oocytes reconstructed with adult cutaneous or fetal fibroblast cell nuclei. Theriogenology.

[49] Song X., Liu Z., He H., Wang J., Li H., Li J., Li F., Jiang Z., Huan Y. (2017). Dnmt1s in donor cells is a barrier to SCNT-mediated DNA methylation reprogramming in pigs. Oncotarget.

[50] Jin L., Guo Q., Zhang G.L., Xing X.X., Xuan M.F., Luo Q.R., Luo Z.B., Wang J.X., Yin X.J., Kang J.D. (2018). The histone deacetylase inhibitor, CI994, improves nuclear reprogramming and in vitro developmental potential of cloned pig embryos. Cell. Reprogram.

[51] Samiec M., Skrzyszowska M. (2018). Intrinsic and extrinsic molecular determinants or modulators for epigenetic remodeling and reprogramming of somatic cell-derived genome in mammalian nuclear-transferred oocytes and resultant embryos. Pol. J. Vet. Sci..

[52] Samiec M., Opiela J., Lipiński D., Romanek J. (2015). Trichostatin A-mediated epigenetic transformation of adult bone marrow-derived mesenchymal stem cells biases the in vitrodevelopmental capability, quality, and pluripotency extent of porcine cloned embryos. Biomed. Res. Int..

[53] Huang J., Zhang H., Yao J., Qin G., Wang F., Wang X., Luo A., Zheng Q., Cao C., Zhao J. (2016). BIX-01294 increases pig cloning efficiency by improving epigenetic reprogramming of somatic cell nuclei. Reproduction.

[54] Samiec M., Skrzyszowska M. (2018). Can reprogramming of overall epigenetic memory and specific parental genomic imprinting memory within donor cell-inherited nuclear genome be a major hindrance for the somatic cell cloning of mammals?–a review. Ann. Anim. Sci..

[55] Takeda K., Tasai M., Iwamoto M., Akita T., Tagami T., Nirasawa K., Hanada H., Onishi A. (2006). Transmission of mitochondrial DNA in pigs and progeny derived from nuclear transfer of Meishan pig fibroblast cells. Mol. Reprod Dev..

[56] Samiec M. (2005). The role of mitochondrial genome (mtDNA) in somatic and embryo cloning of mammals. A review. J Anim Feed Sci..

[57] St John J.C., Moffatt O., D’Souza N. (2005). Aberrant heteroplasmic transmission of mtDNA in cloned pigs arising from double nuclear transfer. Mol. Reprod Dev..

[58] Samiec M. (2005). The effect of mitochondrial genome on architectural remodeling and epigenetic reprogramming of donor cell nuclei in mammalian nuclear transfer-derived embryos. J Anim Feed Sci..

[59] Zhang L., Huang Y., Wu Y., Si J., Huang Y., Jiang Q., Lan G., Guo Y., Jiang H. (2017). Scriptaid upregulates expression of development-related genes, inhibits apoptosis, and improves the development of somatic cell nuclear transfer mini-pig embryos. Cell Reprogram..

[60] Samiec M., Skrzyszowska M. (2013). Assessment of in vitro developmental capacity of porcine nuclear-transferred embryos reconstituted with cumulus oophorus cells undergoing vital diagnostics for apoptosis detection. Ann. Anim. Sci..

[61] Jin L., Guo Q., Zhu H.Y., Xing X.X., Zhang G.L., Xuan M.F., Luo Q.R., Luo Z.B., Wang J.X., Yin X.J. (2017). Quisinostat treatment improves histone acetylation and developmental competence of porcine somatic cell nuclear transfer embryos. Mol. Reprod Dev..

[62] Samiec M., Skrzyszowska M., Opiela J. (2013). Creation of cloned pig embryos using contact-inhibited or serum-starved fibroblast cells analysed intra vitam for apoptosis occurrence. Ann. Anim. Sci..

[63] Lin T., Lee J.E., Oqani R.K., Kim S.Y., Cho E.S., Jeong Y.D., Baek J.J., Jin D.I. (2016). Tauroursodeoxycholic acid improves pre-implantation development of porcine SCNT embryo by endoplasmic reticulum stress inhibition. Reprod. Biol..

[64] Samiec M., Romanek J., Lipiński D., Opiela J. (2019). Expression of pluripotency-related genes is highly dependent on trichostatin A-assisted epigenomic modulation of porcine mesenchymal stem cells analysed for apoptosis and subsequently used for generating cloned embryos. Anim. Sci. J..

[65] Zhang Y., Qu P., Ma X., Qiao F., Ma Y., Qing S., Zhang Y., Wang Y., Cui W. (2018). Tauroursodeoxycholic acid (TUDCA) alleviates endoplasmic reticulum stress of nuclear donor cells under serum starvation. PLoS ONE.

[66] Smemo S., Tena J.J., Kim K.H., Gamazon E.R., Sakabe N.J., Gómez-Marín C., Anease I., Credidio F.L., Sobreira D.R., Wasserman N.F. (2014). Obesity-associated variants within FTO form long range functional connections with IRX3. Nature.

[67] Zhu X.X., Nie J.Y., Quan S.N., Xu H.Y., Yang X.G., Lu Y.Q., Lu K.H., Lu S.S. (2016). In vitro production of cloned and transgenic cloned embryos from Guangxi Huangjiang Xiang pig. Vitro Cell Dev. Biol. Ann..

[68] Nie J.Y., Zhu X.X., Xie B.K., Nong S.Q., Ma Q.Y., Xu H.Y., Yang X.G., Lu Y.Q., Lu K.H., Liao Y.Y. (2016). Successful cloning of an adult breeding boar from the novel Chinese Guike No. 1 swine specialized strain. 3 Biotech..

[69] Zhu X.X., Zhong Y.Z., Ge Y.W., Lu K.H., Lu S.S. (2018). Generation of transgenic-cloned Huanjiang Xiang pigs systemically expressing enhanced green fluorescent protein. Reprod. Domest. Anim..

[70] Wang R., Zhang J.Y., Lu K.H., Lu S.S., Zhu X.X. (2019). Efficient generation of GHR knockout Bama minipig fibroblast cells using CRISPR/Cas9-mediated gene editing. Vitro Cell Dev Biol Ann..

[71] Younis S., Schonke M., Massart J., Hjortebjerg R., Sundstrom E., Gustafson U., Bjornholm M., Krook A., Frystyk J., Zierath J.R. (2017). The ZBED6-IGF2 axis has a major effect on growth of skeletal muscle and internal organs in placental mammals. Proc. Natl. Acad. Sci. USA.

[72] Liu X.F., Liu H.B., Wang M., Li R.Q., Zeng J.H., Mo D., Cong P.Q., Liu X.H., Chen Y.S., He Z.Y. (2018). Disruption of the ZBED6 binding site in intron 3 of IGF2 by CRISPR/Cas9 leads to enhanced muscle development in liang guang small spotted pigs. Transgenic Res..

[73] Zhang S.S., Kim K.H., Rosen A., Smyth J.W., Sakuma R., Delgado-Olguin P., Davis M., Chi N.C., Puviindran V., Gaborit N. (2011). Iroquois homeobox gene 3 establishes fast conduction in the cardiac his-purkinje network. Proc. Natl. Acad. Sci. USA..

[74] Kim K.H., Rosen A., Bruneau B.G., Hui C.C., Backx P.H. (2012). Iroquois homeodomain transcription factors in heart development and function. Circ. Res..

